# Your Co-author Received 150 Citations: Pride, but Not Envy, Mediates the Effect of System-Generated Achievement Messages on Motivation

**DOI:** 10.3389/fpsyg.2018.00628

**Published:** 2018-05-03

**Authors:** Sonja Utz, Nicole L. Muscanell

**Affiliations:** ^1^Social Media Lab, Leibniz-Institut für Wissensmedien, Tübingen, Germany; ^2^Department of Psychology, University of Tübingen, Tübingen, Germany; ^3^Department of Psychology, Penn State York, York, PA, United States

**Keywords:** social comparison, pride, envy, motivation, ResearchGate

## Abstract

ResearchGate, a social network site for academics, prominently displays the achievements of people one follows (“With 150 new reads, X was the most read author from their institute”). The goal of this paper was to examine the emotional and motivational effects of these system-generated messages, thereby extending prior research on envy-evoking status updates on Facebook to a professional context. We also extend the research on social comparisons and more broadly, on emotional responses elicited by social media. Specifically, social media research has largely focused on examining emotional reactions to content that is both generated by and is about others. In this research we directly examine updates generated by the system (ResearchGate) while also directly comparing reactions to updates about others’ achievements with reactions to updates that are about the self—i.e., one’s personal achievements which are also displayed on ResearchGate (“With 150 new reads, you were the most read author from your institute”). Particular attention was paid to the mediating role of envy and pride. The results of our quasi-experimental field study (*n* = 419) showed that the achievements of others elicited envy, whereas personal achievements elicited pride. People exposed to their personal achievements (vs. the achievement of others) showed a higher motivation to work harder. This effect was mediated by pride, but not envy. The theoretical and practical implications of these findings are discussed.

## Introduction

Social network sites (SNS) for researchers such as ResearchGate, Academia.edu or loop are professional networks whose mission is to help their users build a scientific reputation and boost their citations. These networks push information to their users that was either previously not available or needed to be pulled manually from the internet, such as the number of publications and various altmetrics such as views, downloads, or citations. On ResearchGate, users frequently receive system-generated notifications such as “Your co-author’s article reached 150 citations.” In a similar vein, personal successes are also displayed (“Your article reached 150 citations”). What are the emotional and motivational consequences of exposure to such messages? Are academics demotivated when reading about the successes of others or do these messages motivate them to work harder? What role do emotions play in this process? To answer these questions, we conducted a quasi-experiment in the field on the role of system-generated messages on motivation, focusing on envy and pride as potential mediators.

For decades, social comparison research has examined how people react when comparing themselves with others. Various outcomes such as changes in mood, self-esteem, and ability estimates have been studied (see Gerber et al., 2018, for a recent meta-analysis). More recently, there has been increasing attention on social comparison processes on social media (Appel et al., 2016). Much of this research suggests that there is a negative side to seeing what others post. This research has largely focused on Facebook, linking it to envy and depression.

The purpose of our research was threefold. First, we sought to determine whether these social comparison effects would extend to social media that are more professionally oriented. Second, given that people can be exposed to information about others in addition to information about themselves on social media, we sought to determine to what extent emotional reactions may differ based upon the nature of the information. If seeing updates about others provokes envy—what will people feel when they see information about themselves? ResearchGate was an ideal setting to study this question as it exposes individuals to both types of information (what your colleagues have achieved and what you have achieved). Further, this information is always generated by the system thus removing some variability that would occur in self-generated posts and could influence the attribution of whether the colleague expresses authentic or hubristic pride, which would in turn influence whether envy is elicited (Lange and Crusius, 2015).

Third, within the social comparison literature, less attention has been paid to changes in motivation as potential reaction and almost no work has examined the mediating role of discrete emotions triggered by the social comparison [see Nabi and Keblusek (2014), discussed below, for an exception]. Studies on envy have examined the relationship between specific types of envy, i.e., benign (a less hostile form of envy) and malicious envy (a more hostile form of envy) and motivation (van de Ven et al., 2009). For example, benign envy has been found to increase motivation, whereas, malicious envy has been found to be demotivating (van de Ven et al., 2011). However, as mentioned above, it is not clear what reactions people will have when exposed to information about one’s own success—it seems less plausible that this would cause one to be envious and less motivated—thus, we would predict a positive reaction here. Could seeing information about one’s own achievement be motivating? There is research examining the relation between positive emotions on motivation. Specifically, pride, which seems likely to be relevant in the case of one’s own achievements, has been found to increase motivation (Williams and DeSteno, 2008).

Using a naturalistic quasi-experiment, we bring together these lines of research and focus on the mediating role of envy and pride. By examining these together, this should provide a more comprehensive understanding of both the positive and negative emotional effects of system-generated posts frequently encountered on social media platforms such as ResearchGate. We also follow the call by Nabi and Keblusek (2014) to pay more attention to the neglected role of emotions in explaining the effects of social comparison processes on motivation.

### Social Comparisons and Emotions

People compare themselves regularly to others – family members, friends, colleagues, but also to more distant online contacts (Festinger, 1954; Mussweiler et al., 2006). Research on social comparison processes distinguishes upward and downward social comparisons (Festinger, 1954; Buunk et al., 1990; Suls and Wheeler, 2000; Mussweiler, 2003). Upward social comparisons (comparison with those who are more successful, attractive, or somehow better off) frequently trigger the emotion of envy which has been described as an unpleasant and painful emotion in which one feels inferior, resentful, and sometimes even hostile (Smith and Kim, 2007). Alternatively, downward comparisons (comparisons with those who are less successful, attractive, or who are worse off), can lead to positive emotions such as happiness and pride (Weiner, 1985). However, as Buunk et al. (1990) have shown, these relationships are not always so straightforward and both, upward and downward comparisons, can lead to positive affect. Self-esteem or perceived control are important moderators. When it comes to social comparisons on social media, the focus has mainly been on envy.

### Social Comparisons on Social Media

Overall, research on social comparison effects on social media has predominantly focused on Facebook or it’s regional/national predecessors. A review by Verduyn et al. (2017) summarizes the results of these studies. In general, it was shown that passively browsing posts on SNS such as Facebook reduces well-being because it leads to more unfavorable upward social comparisons and evokes feelings of envy.

The research focus on social media envy is not completely surprising as individuals on these sites are often motivated to put their best foot forward, which can lead to an overrepresentation of idealized lives—resulting in feelings that one’s own life as being not-so-perfect. For example, on Facebook, typical envy evoking posts are ones related to vacation and leisure pictures (Krasnova et al., 2013). Physical attractiveness is also a commonly used comparison dimension on social media. That is, people chose attractive photos, and especially on Instagram, these photos are often edited. Deighton-Smith and Bell (2017), for example, did a content analysis of photos posted with the hashtag #fitspiration on Instagram and found that the vast majority of people depicted were low in body fat, and 55% were muscular. Whereas the hashtag #fitspiration suggests that these posts might inspire people, research found downsides of these social media posts such as decreased body satisfaction (Fox and Vendemia, 2016; Hanna et al., 2017) or a higher incidence of disordered eating or compulsive exercise behavior (Holland and Tiggemann, 2017).

Haferkamp and Krämer (2011) compared the effects of upward comparisons with physically attractive others vs. professionally more successful others and found that while all participants showed lower body satisfaction after the comparison with attractive targets, only males were affected by information about targets who were professionally successful. However, they focused on students and used a mock-up SNS. It would thus be premature to conclude that information about professional successes on social media is less threatening. Indeed, a review by Appel et al. (2016) highlights methodological issues such as the use of mock profiles (i.e., individuals are exposed to fictitious information as part of the comparison process), correlational designs, and also points out the contradiction that there are sometimes positive effects stemming from social media use. This latter finding is particularly interesting. Less attention has been on the positive outcomes, and more importantly, what leads to positive psychological outcomes?

The answer may be related (at least in part) to whether people are looking at information about others vs. info about oneself. For example, Toma and Hancock (2013) demonstrated that Facebook use is self-affirming. In their study, participants were more accepting of negative feedback if they had browsed Facebook beforehand. However, this was only true for participants who looked at their own profile vs. looking at a stranger’s profile. The implication is that looking at information about oneself may be positive. We therefore extend prior research by comparing reactions to information about others vs. information about oneself. To our knowledge, no prior study has directly compared the emotions elicited by social media posts that advertise other’s vs. one’s own successes. The focus has been on envy evoked by friends-generated posts or the emotions triggered by receiving likes (Krasnova et al., 2013; Lin and Utz, 2015; Tandoc et al., 2015). Toma and Hancock (2013) have directly compared the effects of looking at one’s own vs. another’s profile, however, it was unclear as to what content individuals actually viewed, and there was no direct comparison of the motivational effects of these two types of posts. We address these issues by asking participants to view real content and report what they viewed, in addition to directly assessing the motivational outcomes.

We argue that research on professional platforms is thus highly needed, especially because prior research has identified differences between platforms. Image-based platforms like Instagram, for example, have a higher potential to ameliorate loneliness than text-based platforms (Pittman and Reich, 2016), and Snapchat elicits higher levels of jealousy than Facebook because of the higher privacy and low persistence of communication (Utz et al., 2015). The affordances of a platform thus influence psychological processes.

Why should the effects of browsing professionally oriented SNS be different from browsing Facebook posts? We argue that the effects might be even stronger on platforms such as ResearchGate for several reasons. First, individuals are more likely to engage in social comparisons when the domain is relevant to them (Smith, 2000). The focus of ResearchGate is quite narrow on academic publications and the related altmetrics and the users are a relatively homogeneous group of academics. The messages are all on number of views, downloads, or citations, and research has shown that the ResearchGate scores correlate positively with traditional indicators of academic success (Thelwall and Kousha, 2015; Yu et al., 2016). The information encountered on ResearchGate is thus highly relevant for academics.

Second, on professional platforms, the focus also tends to be on demonstrating one’s competence and career success. In offline contexts, research has shown that envy is commonly experienced in competitive work-contexts, i.e., in contexts in which people have to compete for resources, bonuses, or promotions (Smith and Kim, 2007; Tai et al., 2012). These findings should also extend to ResearchGate given that academia tends to be a very competitive domain—individuals must compete with others for positions, grant funding, publications, tenure and promotion, academic awards and recognition, and so on (Carson et al., 2013).

Third and most important, many of the posts visible on ResearchGate stem from the system and are not self-generated like on Facebook. This should influence attribution processes and the emotions triggered. A person posting the number of citations received might be perceived as arrogant and expressing hubristic pride which could in turn trigger malicious envy (Lange and Crusius, 2015). System-generated messages are more objective and provide less cues for such person attributions, i.e., might make it harder to devaluate the successes of others as bragging since cues that are more difficult to manipulate are supposed to have a higher warranting value (Tong et al., 2008). There is a positivity norm on Facebook and related platforms (Reinecke and Trepte, 2014; Utz, 2015). Users could thus downplay the effects by attributing posts to a biased selection of their friends. This is more difficult on ResearchGate where an algorithm selects the achievements and presents them as objective facts.

Taken together, there are various reasons to assume that the system-generated posts on ResearchGate trigger social comparisons. In the next paragraphs, we turn to the emotions they might elicit and that we are going to study.

### The Present Research

#### Direct Effects on Emotions and Motivation

Since envy is commonly triggered by social media posts (Verduyn et al., 2017), we first turn to envy, a painful feeling triggered by the superiority of privileged others (Krasnova et al., 2013). Research on envy identified similarity to the comparison target, self-relevance of the comparison dimension, low perceived control, and the feeling that the person does not deserve the object/success as important antecedents of envy (Smith, 2004). The comparison targets on ResearchGate are similar (fellow academics), the domain is highly self-relevant and perceived control might also be low since the high impact journals have high rejection rates and the number of citations can hardly be influenced. Whether people feel that the colleague deserves this amount of attention might vary, but in general, we expect that exposure to the achievements of another researcher triggers envy.

H1: People exposed to the achievement of another researcher experience a higher level of envy than people in the no-achievement group or people in the personal-achievement group.

Within the group exposed to the achievement of another researcher, we can take a closer look at the direction of the comparison. On ResearchGate, there are no explicit downward-comparisons, such as the ones typically used in classical social comparison studies, e.g., “Your co-author has 200 less citations than you.” Instead, the highlighted display of the number of reads or citations always suggests that it is an accomplishment. However, more careful consideration of these indicates that some of these achievements might not be highly impressive, as they could, for example, inform you that a 12-year old paper received 10 citations. This leaves the possibility that some achievements might not be seen as an upward comparison. We do, however, not know whether people deeply process the information given in the achievement or rather heuristically react with envy on the highlighted box in their feed. We therefore pose an open research question on the role of the direction of the comparison.

RQ1: Does the actual direction of comparison (upward vs. downward) affect envy?

ResearchGate also creates a somewhat complex environment when it comes to exploring the effects of messages on its users. Not only are users exposed to the achievements of other researchers, but users also are shown messages about their own personal achievements, e.g., “With 137 reads, you were the most read author from your institution.” Thus, a remaining question is to what extent these personal accomplishments affect emotions and whether this is distinct from the effects of seeing others’ achievements or seeing no achievement at all. Seeing an update about a personal achievement is not directly a social comparison. However, personal achievements could arguable imply a downward social comparison; the fact that the system has singled out your achievement may automatically imply that you are doing better than others.

We examine pride as emotion that could be elicited by an update about one’s own achievement. Pride is a positive emotion in reaction to a positive outcome (Kornilaki and Chlouverakis, 2004). Situations that trigger pride usually also trigger happiness (but not necessarily the other way around). To experience pride, people must feel responsible for the positive outcome and the outcome must surpass a standard (Kornilaki and Chlouverakis, 2004). We assume that academics usually attribute the success of their publications to themselves. The public display of the achievement signals that the outcome is worth to be highlighted. Webster et al. (2003) showed that pride was highest when praise was public and explicit social comparison information was given. Achievements on ResearchGate are public within the community of fellow researchers. They also often contain explicit social comparison information (e.g., “most read from your institution”). We therefore expect that people exposed to their personal achievements experience pride.

H2: People exposed to their personal achievement show higher levels of pride than people in the no-achievement or the other-achievement group.

Next, we turn to the question on how these system-generated achievements affect motivation.

Research on the motivating role of upward or downward social comparisons has revealed mixed results; assimilation and contrast effects have been found, also depending on the self-relevance of the comparison dimension and the similarity of the comparison target (Smith, 2000; Mussweiler, 2003). We focus on moving-up motivation, which is motivation that induces people to work harder and not on push-down motivation focusing on derogating the target (van de Ven et al., 2009). As said above, the achievements on ResearchGate focus narrowly on publications and the various altmetrics related to them, a highly self-relevant domain for academics. Lockwood and Kunda (1997) have shown that upward-comparisons on self-relevant domains motivate and inspire, but only if the performance seems attainable. In a similar vein, Mussweiler et al. (2006) have shown that water polo players estimate their own athletic abilities as lower when compared to an extremely superior standard than when compared to a moderately superior standard. We do not know whether ResearchGate predominantly displays the achievements of people of the same career stage or of superior colleagues. Assuming that there is at least a mix of comparisons with moderately and extremely superior colleagues, some of the achievements of other researchers might motivate users, whereas other might demotivate them.

The situation is clearer for personal achievements. Due to the highlighted display and framing as achievement, they could be seen as an instance of praise, and research on praise has found that praise on a relevant task usually increases motivation (Delin and Baumeister, 1994). We thus expect that people exposed to their personal achievements are on average more motivated than people exposed to the achievements of others.

H3: Moving-up motivation is higher in the personal achievement group than in the other’s achievement group.

#### The Mediating Role of Emotions

Nabi and Keblusek (2014) argued that it is not the gap between oneself and the comparison target that increases motivation, but the discrete emotion elicited by perceiving this gap. Taking the mediating role of emotions into account might therefore help to clarify the inconsistent findings of prior research.

Emotions differ in their associated action tendencies (Frijda et al., 1989). Anger for example is associated with attacking, whereas sadness is associated with inaction and withdrawal (Lazarus, 1991). Nabi and Keblusek (2014) focused on envy, hope, and sadness as likely responses to upward social comparisons and, importantly, as mediators explaining the relationship between social comparison and motivation. They studied the effects of these emotions in the context of comparison with media figures, more specifically, protagonists in reality television cosmetic surgery programs. Envy is associated with the action tendency of seeking and possessing and therefore was predicted to motivate people toward the goal of undergoing cosmetic surgery. Sadness is associated with inaction, so no motivating effect was expected. In line with these predictions, envy predicted increased motivation to undergo cosmetic surgery.

However, it remains unclear to what extent these results on social comparisons with media figures will hold for social comparisons on SNS. Lim and Yang (2015) focused on envy and shame elicited by social comparisons on SNS and found that envy mediated the effect of social comparisons on intention to switch to another platform. Envy might therefore also be demotivating.

Research on workplace envy also has led to inconsistent results. Schaubroeck and Lam (2004) found that people who have been rejected for a promotion showed higher performance 7 months later. This pattern would be in line with the idea that envy motivates people to work harder. However, Duffy and Shaw (2000) found reduced team performance as consequence of workplace envy, indicating that envy might also be negatively related to moving-up motivation. It could thus be that envy further demotivates people in a competitive context in which demotivating rejections are a common experience (Carson et al., 2013). Researchers have also found that it is the benign envy (and not malicious envy) triggered by upward comparisons that leads to higher study motivation and better performance on intelligence and creativity tests (van de Ven et al., 2011). Rentzsch et al., 2015 found that envy mediates effects of low self-esteem on hostile tendencies after upward comparisons. They did not differentiate between benign and malicious envy, but since malicious envy has been found to be related to pulling-down motivation (van de Ven et al., 2011), this finding suggests that lay-persons associate general envy items with malicious envy.

Due to the nature of our natural field experiment, we could not disentangle between benign and malicious envy as a reaction to the achievement because we did not assess these emotions in the no achievement and the personal achievement groups. The benign and malicious envy items would require a comparison target (e.g., “I feel ill will toward the person with the achievement”). Thus, we could not include this in the no achievement because there was no comparison target, and it would not make sense in the personal achievement group (e.g., “I feel ill will toward myself”). Although researchers distinguish between benign and malicious envy, lay-persons, typically view it as being one broad construct and perceive it as negative (van de Ven et al., 2009). It is thus likely that participants who answer a general envy item may have malicious envy in mind. Since the findings of van de Ven et al. (2011) on a potential relationship between malicious envy and moving-up motivation were mixed, we formulated a research question:

RQ2: Does envy mediate the effect of social comparison on moving-up motivation?

However, to add to the value of the conceptual distinction between benign and malicious envy, we measured these as traits and expected to replicate the findings of van de Ven et al. (2011) at the trait level.

H4: Trait benign envy is positively related to moving-up motivation.

Pride could also increase moving-up motivation since it is characterized by approach tendencies. To our knowledge, no studies have tested the mediating role of pride on motivation, although some authors speculated that positive affect might be a mediator (Delin and Baumeister, 1994). There are, however, studies showing that pride can increase perseverance (Williams and DeSteno, 2008), goal importance (Hofmann and Fisher, 2012) and work engagement (Lee et al., 2017). It might thus be that personal achievements lead to higher motivation via a mediating role of pride.

Based on these findings, we predict a mediating role of pride:

H5: Pride mediates the effect of personal achievements (vs. other’s achievements) on moving-up motivation.

## Materials and Methods

### Participants and Procedure

The study was conducted with Qualtrics Online Survey Solutions. Invitations were spread via Twitter, Facebook and academic mailing lists. The study was approved by the ethical board of Leibniz-Institut für Wissensmedien. The first page stated that participants need to have an account on ResearchGate to be eligible. Participants gave their consent by clicking the appropriate box on the informed consent page before starting with the actual survey. Participants were asked to open their ResearchGate account and indicate whether there was an achievement (an example was given) among their recent updates. Participants were told to look only for recent updates that can be accessed without too much scrolling. Participants who reported an achievement were asked whether it was their personal achievement or the achievement of another person. All participants received questions on their momentary feelings (see below) after these general achievement checks. People who saw an achievement also received questions on motivation. When the achievement was from someone else, questions on the relationship to the other person and the direction of the comparison were asked. We also assessed personality characteristics. At the end, respondents could indicate their sex, age and career level.

The survey went online on November, 12, 2015. We kept the survey very short because we hoped it would go viral on Twitter, but this did not happen. When we announced it in mailing lists of our own network (psychology and communication science), we saw a clear increase in participants shortly after. Further attempts to collect additional data via emails to researchers and invitations via the Qualtrics panel feature resulted in response rates of less than 1%. We closed the survey on December 7, 2015 because we also noticed that ResearchGate has introduced a “like” feature on the achievements which could affect social comparison processes. After a first preliminary analysis of the number of achievements and the prevalence of upward and downward comparisons, we decided to add a more specific item for comparison direction and a few questions on the use of the new like button and extended the data collection in 2016.

The questions on sex and age were voluntary, but at data collection time 1, 218 (102 females, 116 males) people out of the 328 people who clicked on the start page indicated their sex. At data collection time 2, a similar distribution (92 females, 109 males; out of 391 who clicked on the start page; one person excluded because he/she asked for deletion of the data and indicated that he/she just clicked through to see the questions) was reported. The two subsamples also had a similar age; *M* = 38.86 (*SD* = 11.28) for subsample 1 and *M* = 36.40 (*SD* = 8.38) for subsample 2. Both samples covered the whole range of career levels (2.8%/2% master students, 16.2%/28.9% Ph.D. students, 25%/27.4% post-docs, 21.3%/20.4% assistant professors, 15.7%/12.4% associate professors, 19%/9% full professors).

### Measures

#### ResearchGate Achievements

The questions for ResearchGate achievements was a simple yes/no question—“did you see an achievement in your newsfeed?” People who chose “yes” received a follow-up question on whether it was their personal achievement or the achievement of someone else.

#### Emotions

Respondents indicated on self-developed 7-point semantic differentials whether they felt ashamed-proud (*M* = 4.42, *SD* = 0.98) or content-envious (*M* = 3.16, *SD* = 1.28) right now. We also assessed the following additional emotions to strengthen the claim that envy and pride whereas general negative and affective affect drive the results: unhappy–happy (*M* = 4.66, *SD* = 1.18), cheerful-depressed (*M* = 3.42, *SD* = 1.12), calm–angry (*M* = 2.74, *SD* = 1.26). The results including all five emotions are reported in the Supplementary Materials.

#### Moving-Up Motivation

Respondents who saw an achievement indicated their agreement to the statements “I feel inspired” and “I want to try to work harder” on 7-point scales ranging from 1 = strongly disagree to 7 = “strongly agree.” These two items were adapted from van de Ven et al. (2009) and formed a reliable scale, α = 0.72 (*M* = 4.15, *SD* = 1.21). After the introduction of the like button, we also assessed motivation in the no achievement group.

#### Comparison Direction

Respondents indicated how the person with the achievement performed in their career in relation to themselves on a five-point scale ranging from 1 = a lot worse than me to 5 = a lot better than me (*M* = 4.12, *SD* = 1.08). At time 2, a more specific item was added: “How would you judge this specific performance, compared to your own performance and considering relevant constraints such as the publication date of an article in case it is an achievement on number of citations?” Answers were given on a 7-point Likert-scale ranging from “worse than me” to “better than me” (*M* = 5.37, *SD* = 1.40).

#### Trait Malicious and Benign Envy

Trait malicious and benign envy were measured each with two items from the benign and malicious envy scale (BeMaS) by Lange and Crusius (2015). An example item for benign envy is “If I notice that another person is better than me, I try to improve myself.” An example for malicious envy is “I feel ill will toward people I envy.” Answers were given on six-point scales ranging from 1 = strongly disagree to 6 = strongly agree (benign envy: *M* = 3.68, *SD* = 1.07, α = 0.75; malicious envy: *M* = 2.34, *SD* = 1.06, α = 0.82).

#### Additional Measures

In the other’s achievement group, we included some additional items about the target (location, co-authorship, closeness, identification with the target). At time 1, we also assessed perceived fairness of achievements, and a few items on ResearchGate usage. At time 2, we asked about the nature of the achievements, including whether the achievement was about citations, downloads, and the exact number of citations/downloads or whether it was a relative (“most downloaded/read from the institute”) or absolute (“150 citations”) achievement. We also asked people whether they shared achievements on other social media. Respondents could also comment on the study. Social comparison orientation was also measured, but did not alter the effects.

These variables are not included in the current paper; the results on the use of the like button are reported in the Supplementary Materials; additional information is available from the corresponding author.

We included all studies in the paper and mentioned all measures in the “Materials and Methods” section.

The correlations between the emotions and motivation are displayed in **Table 1**.

**Table 1:** Correlations of emotions and motivation.

	1	2	3	4
(1) Pride	-			
(2) Envy	-0.42^∗∗∗^	-		
(3) Trait benign envy	0.13^∗∗^	0.08	-	
(4) Trait malicious envy	-0.12^∗^	0.33^∗∗^	0.29^∗∗^	-
(5) Motivation	0.44^∗∗∗^	-0.28^∗∗^	0.26^∗∗^	-0.12^∗^

*^∗^p < 0.05, ^∗∗^p < 0.01, ^∗∗∗^p < 0.001.*

## Results

### Descriptive Results – Prevalence of Achievements

Since we use ResearchGate as setting for a quasi-experiment in the field, and features and algorithms behind social media platforms change frequently, it is important for interpretation and potential replication of results to describe them at the moment of the data collection. We therefore report first descriptive data on the prevalence of achievements and the direction of comparisons.

The question on the achievements was completed by 482 people (248 at time 1, 234 at time 2). Some of these dropped out during the study, but we decided to report the results based on the valid cases; *n*s therefore differ slightly between analyses. Especially for the first items on the type of achievement, this procedure should give a more accurate picture. The vast majority of respondents (85.5% at time 1, 73.5% at time 2) reported seeing an achievement. Roughly one third (30.7% at time 1; 31.4% at time 2) of those exposed to an achievement saw their personal achievement, whereas the remaining were exposed to the achievement of someone else. The majority of these comparisons were upward comparisons. A vast majority (48.9% at time 1, 52.8% at time 2) reported to have seen the achievement of a person performing a lot better than them. Only 1.5%/3.8% (at times 1 and 2, respectively) indicated that they have been shown the achievement of a person performing a lot worse, 8.9%/3.8% saw a person performing worse, 17.8%/18.9% saw a person performing on the same level, and 23%/20.8% saw a person performing a little bit better. The more fine-grained measure on the achievement level at time 2 also indicated a strong prevalence of upward comparisons; the mean was 5.37 (*SD* = 1.4) on a scale ranging from 1 “worse than me” to 7 “better than me.” This variable was negatively related to career stage, *r*(105) = -0.28, *p* < 0.01 (Spearman-Rho). Thus, especially people in earlier career stages are exposed to upward comparisons. However, even full professors reported mainly upward comparisons (*M* = 4.5, *SD* = 1.69).

These descriptive results have implications for the main analyses. The cell sizes are unequal (*n* = 94 in the no achievement group, *n* = 282 in the other’s achievement group and *n* = 108 in the personal achievement group) and there are almost no downward-comparisons. We therefore report Pillai’s trace in the MANOVA because it is more robust in cases of unequal cell size.

Time of data collection (before/after introduction of the like button) was added as potential moderator in the following analyses. Since it did not show any moderating effect and for reasons of brevity, the results of the merged data set instead of repeating almost identical results are reported in the paper (see the Supplementary Materials for the analysis of the time 1 data).

### Effects on Emotions

A MANOVA with achievement group (none, personal, other’s) and time of data collection (before vs. after the introduction of the like button) as the independent variables and pride and envy as dependent variables revealed only a significant multivariate effect of achievement group, Pillai’s trace = 0.09, *F*(4,896) = 10.61, *p* < 0.001, $\eta_{p}^{2}$ = 0.05. The effect of time of data collection, Pillai’s trace = 0.002, *F*(2,447) = 0.35, *p* = 0.702, $\eta_{p}^{2}$ = 0.003 and the interaction effect, Pillai’s trace = 0.003, *F*(4,896) = 0.36, *p* = 0.840, $\eta_{p}^{2}$ = 0.002, were not significant. As can be seen in **Table 2**, univariate tests showed significant effects for both emotions.

**Table 2:** Means (and standard deviations) of emotions depending as a function of achievement group.

	None	Other	Personal	*F*	*p*	$\eta_{p}^{2}$
Pride	4.17_a_ (0.88)	4.33_a_ (0.93)	4.86_b_ (1.04)	16.24	<0.001	0.068
Envy	2.96_ab_ (1.20)	3.34_a_ (1.34)	2.93_b_ (1.13)	5.04	0.007	0.022

*Means within a row not sharing the same subscript letters are significantly different at p < 0.05 (Bonferroni-corrected comparisons).*

With regard to H1, we found that respondents experienced more envy when someone else had reached an achievement than when they themselves reached an achievement; here, the control group fell non-significantly between the other two groups. However, a planned comparison with a Welch test (controlling for unequal cell sizes and inhomogeneous variances) revealed that envy was higher in the others’ achievement group than in the two other groups, *Welch F*(1,449.01) = 11.08, *p* < 0.001. In line with H2, people who were exposed to a personal achievement felt more proud than people who saw no achievement or the achievement of another person.

In RQ1 we asked whether comparison direction influenced emotions. This can only be analyzed within the subgroup of people who have seen the achievement of another person. Due to the skewed distribution and the low prevalence of downward comparisons, we decided not to dichotomize the sample into downward and upward comparisons, but to look at the correlations with the items for comparison direction. Envy was not significantly correlated to the item on performance of the comparison target at the career level, Spearmans Rho(241) = 0.09, *ns*. At time 2, we had included a more specific item focusing on the displayed achievement. This item correlated significantly with envy, Spearmans Rho(110) = 0.22, *p* = 0.02, such that increased feelings of envy were associated with increased perceptions that another person was performing better than the participant. Thus, we found some evidence that perceived upward comparisons are related to higher levels of envy.

### Effects on Moving-Up Motivation

A univariate analysis of variance with source of achievement as independent variable revealed a significant effect of source, *F*(1,356) = 7.55, *p* = 0.006, $\eta_{p}^{2}$ = 0.021. As predicted by H3, participants were more motivated when they were exposed to their personal achievement (*M* = 4.39, *SD* = 1.28) than when they were exposed to someone else’s achievement (*M* = 4.01, *SD* = 1.17). Neither the main effect of time nor the interaction effect were significant, both *F*s < 1, ns.

### Mediation Effects

**FIGURE 1:**
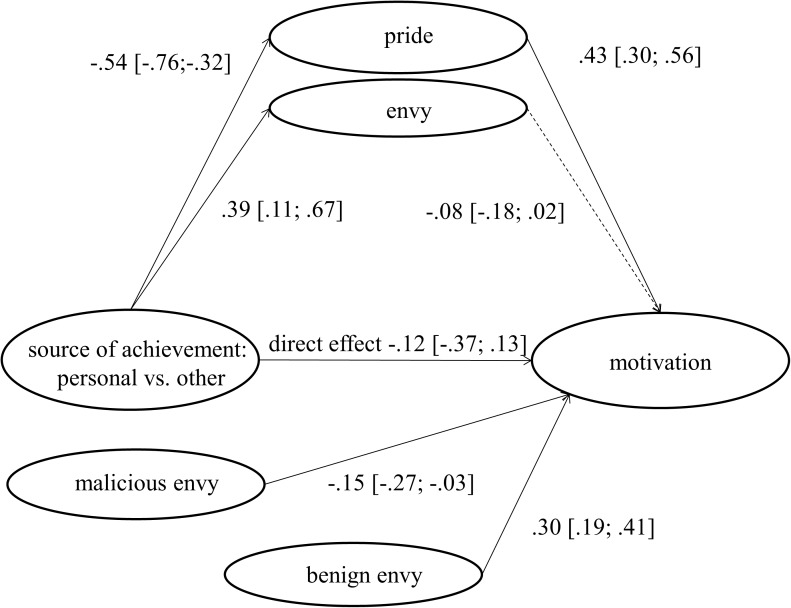
Effects of achievement source on emotions and motivation, controlling for effects of trait benign and malicious envy.

To test whether the emotions mediate the effects of source of achievement on motivation we used Process by Hayes (2013). We used *model 4*, a model with several parallel mediators. Source of comparison was used as the independent variable, motivation as the dependent variable. The two emotions were entered as mediators; trait benign and malicious envy were included as covariates (see **Figure 1**). We opted for 10.000 bootstrapping samples.

The direct effect of achievement source (personal vs. other) on motivation was not significant anymore once controlling for emotions, -0.12, *SE* = 0.13, 95% CI [-0.37; 0.13]. More important, there was a significant indirect effect via pride, -0.23, *SE* = 0.06, 95% CI [-0.38; -0.12]. Respondents experienced more pride in the personal achievement condition which in turn increased their motivation. H5 is thus supported. There was no significant indirect effect via envy, -0.03, *SE* = 0.03, 95% CI [-0.10; 0.01]. The answer to the RQ2 is thus that general envy is unrelated to motivation. In line with H4, benign envy was positively related to motivation, 0.30, *SE* = 0.06, 95% CI [0.19; 0.41]. Malicious envy in contrast was negatively related to motivation, -0.15, *SE* = 0.06, 95% CI [-0.27; -0.03]. The model also revealed that trait benign envy was positively related to pride, 0.16, *SE* = 0.05, 95% CI [0.06; 0.25]), whereas trait malicious envy was negatively related to pride, -0.14, *SE* = 0.05, 95% CI [-0.25; 0.04]). Interestingly, only trait malicious envy was related to state envy, 0.38, *SE* = 0.07, 95% CI [0.25; 0.52]), indicating that the single-item envy measure indeed mainly taps into malicious envy.

## Discussion

The aim of this study was to use the natural media setting of ResearchGate to extend research on social comparison processes and envy-evoking social media posts to the professional domain. The achievements of others elicited envy, whereas personal achievements elicited pride. Being exposed to personal achievements resulted in a higher work motivation than being exposed to the achievements of others. Pride but not envy mediated the effect of achievement source on motivation.

### Theoretical Implications

These results have several theoretical implications. First, we contribute to the literature on social comparison processes on social media by showing that not only friend-generated status updates can elicit envy (Krasnova et al., 2013; Kross et al., 2013; Tandoc et al., 2015) but also system-generated messages on professional platforms. System-generated information has a higher warranting value (Tong et al., 2008) that makes it harder to discount the achievement of another researcher by attributing it to their bragging personality or as an instance of hubristic pride (Lange and Crusius, 2015). Hubristic pride triggers malicious envy, but our findings indicate that also rather neutrally formulated system-generated messages can elicit malicious envy. Prior research has also shown that visiting the personal Facebook profile can be self-affirming (Toma and Hancock, 2013). Our results show that also system-generated messages on personal achievements can have positive effects on emotions and motivation. Removing the bragging component might also make it easier to be proud on own achievements. Future research should systematically compare self-, friend- and system-generated posts. Furthermore, we extend the literature by directly comparing reactions to information about oneself vs. others. Similar to Toma and Hancock (2013), our findings suggest that social media can produce positive outcomes, particularly when being exposed to information about oneself. One implication is that users who are other-focused and are therefore more inclined to focus on information about others may be particularly prone to the more negative effects of social media, including envy.

Second, we also extend prior research on envy evoked by social media posts to the professional context. Prior research has shown weaker results for the professional domain than for physical attractiveness (Haferkamp and Krämer, 2011), but this was probably due to the not-yet working student sample and the mock-up network used in this study. We found academics do experience envy when confronted with the success of fellow academics. Our findings are thus in line with studies on workplace envy (Duffy and Shaw, 2000) – at least on the correlational level where we found a negative relationship between envy and motivation, but also at the trait level.

Third, our research sheds more light on the mediating role of discrete emotions in social comparison processes. We found that pride mediated the effect of achievements on motivation. The link between pride and motivation has been demonstrated in related domains (Williams and DeSteno, 2008; Hofmann and Fisher, 2012; Lee et al., 2017), but the full mediation from system-generated social media posts on personal achievements via pride on motivation has not been shown before. Since pride was significantly higher in the own achievement group than in the other two conditions, we interpret it as pride stemming from one’s own achievement. Pride was descriptively somewhat higher in the other’s achievement condition than in the no achievement condition – an interesting question for future research would be whether pride toward another person (e.g., a former Ph.D. student) might also have a motivating function. We assume that this would mainly happen for people who feel close to each other due to interpersonal friendships or social identification, so that the self-other overlap is high and pride on the other person also reflects on the target.

In contrast to Nabi and Keblusek (2014), we did not find a motivating effect of envy. On the correlational level, there was even a negative relationship between envy and motivation (see **Table 2**). It is thus important to consider contextual differences when examining the mediating role of emotions. Nabi and Keblusek (2014) examined the effects of envy on the motivation to undergo cosmetic surgery. The beauty make-over programs studied by them promise a quick solution to a problem which might increase perceived control. In the domain of academic publications, no such quick and easy solution is available and perceived control is lower; this might be the reason why envy tends even to demotivate people (on the correlational level) in this context. Perceived control might thus not only be an important determinant of envy (Smith, 2004), but also influence the motivational effects of envy. Another explanation of the divergent findings could lie in the different forms of envy – prior research has shown that benign envy is the leveling up motivation whereas malicious envy results in pulling down the comparison target. Nabi and Keblusek used also general items (envious, jealous), so it is unclear whether they assessed benign or malicious envy. The correlations of our state envy item with trait benign and malicious envy indicate that it has tapped more into malicious envy. The negative correlation with motivation also supports this interpretation (see Rentzsch et al., 2015, for a similar pattern). The inconsistent findings on the role of envy in work contexts (Duffy and Shaw, 2000; Schaubroeck and Lam, 2004; Tai et al., 2012) might be due to the fact that these studies often did not differentiate between different types of envy.

Our results also contribute to research on benign vs. malicious envy. On the trait level, we were able to replicate the motivating effect of benign envy (van de Ven et al., 2011). In contrast to van de Ven et al. (2011), we also found a significant, albeit weaker, negative effect of malicious envy on motivation. Trait malicious envy was also related to lower levels of pride indicating that chronic malicious envy has negative effects in several respects. In case of work-related achievements, it might trigger a vicious circle. People scoring high on malicious envy do not only experience higher levels of envy, but also lower levels of pride, which in turn makes them less motivated to work harder which might result in lower performance levels and more unfavorable comparisons in the future. Longitudinal studies could explore the long-term consequences of malicious envy.

In general, our results show that it is important to take the domain and the type of comparison processes into account when developing a theory on the mediating role of discrete emotions in social comparison processes. Future research could build on our findings and explore whether the relationship with the comparison target (e.g., close colleague vs. competitor in job applications) moderates the effects within the other’s achievement group. As discussed above, pride might be higher for close others. The relationship with the target could also influence which type of envy is triggered. Research on envy on Facebook has shown that closeness is positively related to benign envy (Lin and Utz, 2015). Malicious envy might be triggered/be stronger when the other is an opponent.

### Practical Implications

Our findings also have practical implications. First, they show that the algorithms determine the type of emotion experienced by the users. People saw their personal achievement in about a third of the cases. One could argue that ResearchGate triggers envy because it predominantly exposes its users to achievements of much better performing others. This could explain why Muscanell and Utz (2017) found that ResearchGate users report higher levels of stress than non-users. On the other hand, considering that most users follow at least several people, a base rate of 30% personal achievements is disproportionally high and one could argue that ResearchGate tries to elicit positive affect, in this case, it can trigger pride and motivate its users. Kramer et al. (2014) showed that subtle changes in the Facebook news feed affect emotions; our results show that this holds also for the proportion of personal vs. other’s achievements and that the emotions in turn affect motivation.

These results also have practical implications for other professional platforms such as enterprise social media. Hitherto, the research focus has been on their potential for organizational knowledge sharing (Leonardi, 2015), but the present results show that these platforms might also create unintended social dynamics. Whereas the public display of an achievement could motivate the person who accomplished it, the same update might demotivate several coworkers. Companies should thus be very careful when incorporating system-generated information on the work performance of their employees.

For academics, our results imply that it might be wise to switch off notifications on the achievements of other researchers and focus more on personal achievements. Raising awareness that system-generated information is not always neutral, but often biased, might also help to dampen negative emotional effects of social comparisons.

### Limitations and Strengths

Before closing, we would like to briefly discuss the limitations and strengths of our study. A limitation is that the sample was not representative for all academic fields; communication scientists and psychologists are likely to be overrepresented, considering the number of hits shortly after sending the invitation to respective mailing lists in these disciplines. However, since social comparison is a fundamental process (Festinger, 1954; Mussweiler, 2003; Gerber et al., 2018) there is no theoretical reason to expect that social scientists would react differently to social comparisons than researchers from other disciplines; therefore, we are confident that our results would also hold for other disciplines. Another limitation is that we assessed emotions only with one item each. We deliberately kept the survey very short in order to maximize the chances the individuals would complete the entire survey although they were required to log into ResearchGate and browse their feed. We were interested in the emotions lay-persons experience when exposed to system-generated statements; in this case, single-item measures can be adequate (Weidman et al., 2017). One could argue that choosing “content” as opposite label of the “envious” semantic differential was not the best choice. “Content” shows up as antonym of envious in antonym finders such as www.thesaurus.com; we therefore believed that lay-persons do perceive this as an adequate scale anchor. Although the positive correlation with trait malicious envy and the pattern of means (higher only in the other achievement condition) indicate that this item might have captured state envy, future research should use several items focusing only on envy.

We only measured motivation to work harder, but we have no objective data on actual productivity. Examining whether motivation translates into a higher scientific output would be an interesting extension for future work. We also did not assess much background information or detailed measures of ResearchGate feature use. We used ResearchGate as a natural setting; it is therefore unclear whether the results can also be generalized to different social media platforms. We expect that they can be generalized to other professional platforms, be it public networks such as LinkedIn or company-intern enterprise social networks. ResearchGate might also change/have changed the algorithm meanwhile; so the findings on the prevalence of the different types of achievements might not be reproducible. The emotional and motivational effects should, however, be stable. The quasi-experimental nature of our study might be considered as a limitation as it resulted in unequal cell sizes and made random allocation of participants to achievement conditions impossible.

This design is at the same time also a major strength of our study – it has high external validity because it took place in the field. Research on social comparison effects often requires people to recall an experience of a comparison situation, and faces therefore problems of biased memory. Further, research on social comparisons on social media has often utilized mock profiles or correlational designs that ask participations to recollect their general behaviors. In our study, participants made a real comparison or viewed real information about themselves—they were asked to view actual content in the moment (not to recall an average from past usage). Additionally, past research largely compared downward- and upward comparisons, but neglected personal achievements as more implicit downward comparisons, focusing thus only on one part of reality. By using the achievements displayed by ResearchGate as quasi-experimental conditions, we brought together research on social comparison, envy and praise. The introduction of the like-button during our data collection introduced another quasi-experimental variation and demonstrates the generalizability of the effects across system changes. Although we did not get as many participants as we aimed for, the sample size is still enough to reach a power of 0.80 for our central mediation analyses (Fritz and Mackinnon, 2007).

Taken together, we have shown that system-generated information on professional SNS triggers emotions and affects motivation. As ResearchGate predominantly shows the achievements of others, it may often trigger envy. Our results also stress the important role of discrete emotions in social comparison processes. Feeling pride in response to personal achievements is the main driver of increased motivation.

## Ethics Statement

This study was carried out in accordance with the recommendations of the DGPS (German Society for Psychology and APA). The protocol was approved by the local ethics board of the Leibniz-Institut für Wissensmedien. All subjects gave online informed consent in accordance with the Declaration of Helsinki.

## Author Contributions

SU and NM jointly designed the study, recruited participants, and wrote the manuscript. SU analyzed the data.

## Conflict of Interest Statement

The authors declare that the research was conducted in the absence of any commercial or financial relationships that could be construed as a potential conflict of interest.
