# Supplementary material for: Your Co-author Received 150 Citations: Pride, but Not Envy, Mediates the Effect of System-Generated Achievement Messages on Motivation
Source: Front Psychol. 2018 May 3;9:628. doi: 10.3389/fpsyg.2018.00628 (PMC5946016; doi:10.3389/fpsyg.2018.00628)
Supplement: Supplementary file 1 [file Presentation_1.pdf]

Supplement

Content

Analysis with all five measured emotions

- Correlations
- MANOVA with emotions as dependent variable
- Process model including all five emotions as mediators

Data collected before the introduction of the like button (time 1 sample)

- MANOVA with pride and envy as dependent variable
- ANOVA with moving-up motivation as dependent variable
- Process model including all five emotions as mediators

Data collected after the introduction of the like button (time 2 sample)

- ANOVA with moving-up motivation as dependent variable (measured also in the no achievement condition)

Exploratory analysis

- Use of the like-button

Analysis with all five measured emotions

Table 1: Correlations of emotions and motivation

	1	2	3	4	5
1. happiness					
2. pride	.62***				
3. depression	-.71***	-.56***			
4. anger	-.43***	-.35***	.46***		
5. envy	-.53***	-.42***	.59***	.64***	
6. motivation	.42***	.44**	-.41***	-.21***	-.28***

Note: * p < .05, ** p < .01, *** p < .001

MANOVA with emotions as dependent variable

A MANOVA with achievement group (none, personal, other's) and time of data collection (before vs. after the introduction of the like button) as the independent variables and the five emotions as dependent variables revealed only a significant multivariate effect of achievement group, Pillai's trace = .10, $F(10,890) = 4.78$, $p < .001$, $\eta_p^2 = .05$. The effect of time of data collection, Pillai's trace = .003,

F(5,444) = 0.26, p = .935, η_p^2= .003 and the interaction effect, Pillai's trace = .014, F(10,896) = 0.643, p = .777, η_p^2= .007, were not significant. As can be seen in Table 2, univariate tests showed significant effects for four of the five emotions.

Table 2: Means (and standard deviations) of emotions depending as a function of achievement group

	None	Other	Personal	F	p	η^2_{part}
happiness	4.45_a (1.19)	4.60_a (1.19)	4.98_b (1.10)	5.74	.003	.025
pride	4.17_a (.88)	4.33_a (.93)	4.86_b (1.04)	16.24	<.001	.068
depression	3.53_a (1.14)	3.49_a (1.07)	3.19_a (1.19)	3.04	.049	.013
anger	2.69_a (1.34)	2.79_a (1.24)	2.66_a (1.25)	0.48	.621	.002
envy	2.96_{ab} (1.20)	3.34_a (1.34)	2.93_b (1.13)	5.04	.007	.022

Note: Means within a row not sharing the same subscript are significantly different at p < .05 (Bonferroni-corrected comparisons)

With regard to H1, we found that respondents experienced more envy when someone else had reached an achievement than when they themselves reached an achievement; here, the control group fell non-significantly between the other two groups. However, a planned comparison with a Welsh's t-test (controlling for unequal cell sizes and inhomogeneous variances) revealed that envy was higher in the others' achievement group than in the two other groups, *Welshs* F(1, 449.01) = 11.08, p < .001. In line with H2, people who were exposed to a personal achievement felt more proud (and also happier) than people who saw no achievement or the achievement of another person. No significant effects were found for anger. Although the main effect on depression was significant, the pairwise comparison between personal and other's achievement reached only marginal significance.

Process model

To test whether the emotions mediate the effects of source of achievement on motivation we used Process by Hayes (2013). We used *model 4*, a model with several parallel mediators. Testing the role of all five discrete motivations simultaneously is a stronger test of the proposed underlying processes, especially since the emotions are correlated with each other (see Table 1). Source of comparison was used as the independent variable, motivation as the dependent variable. The five emotions were entered as mediators. Benign and malicious envy were entered as covariates. We opted for 10.000 bootstrapping samples.

PROCESS OUTPUT (variables have been renamed)

Run MATRIX procedure:

```
************* PROCESS Procedure for SPSS Release 2.16.3 *****************

          Written by Andrew F. Hayes, Ph.D.       www.afhayes.com

    Documentation available in Hayes (2013). www.guilford.com/p/hayes3

**************************************************************************

Model = 4

    Y = motivati

    X = who

   M1 = pride

   M2 = envy
```

M3 = happiness

M4 = depression

M5 = anger

Statistical Controls:

CONTROL= benign malicious (process only takes 8 letters: stands for trait benign envy and trait malicious envy)

Sample size

342

****

Outcome: pride

Model Summary

R	R-sq	MSE	F	df1	df2	p
.3106	.0965	.9235	12.0327	3.0000	338.0000	.0000

Model

	coeff	se	t	p	LLCI	ULCI
constant	5.1762	.2685	19.2760	.0000	4.6480	5.7044
source	-.5368	.1125	-4.7735	.0000	-.7580	-.3156
benign	.1562	.0497	3.1416	.0018	.0584	.2540
maliciou	-.1443	.0520	-2.7768	.0058	-.2465	-.0421

****

Outcome: envy

Model Summary

R	R-sq	MSE	F	df1	df2	p
.3425	.1173	1.5040	14.9691	3.0000	338.0000	.0000

Model

	coeff	se	t	p	LLCI	ULCI
constant	1.5961	.3427	4.6578	.0000	.9221	2.2701

source	.3894	.1435	2.7132	.0070	.1071	.6716
benign	.0117	.0634	.1841	.8541	-.1131	.1365
maliciou	.3848	.0663	5.8028	.0000	.2544	.5153

****

Outcome: happiness

Model Summary

R	R-sq	MSE	F	df1	df2	p
.2697	.0727	1.2890	8.8356	3.0000	338.0000	.0000

Model

	coeff	se	t	p	LLCI	ULCI
constant	5.4920	.3172	17.3120	.0000	4.8680	6.1160
source	-.3496	.1329	-2.6318	.0089	-.6110	-.0883
benign	.1247	.0587	2.1232	.0345	.0092	.2402
maliciou	-.2642	.0614	-4.3038	.0000	-.3850	-.1435

****

Outcome: depression

Model Summary

R	R-sq	MSE	F	df1	df2	p
.2910	.0847	1.1612	10.4269	3.0000	338.0000	.0000

Model

	coeff	se	t	p	LLCI	ULCI
constant	2.7762	.3011	9.2200	.0000	2.1839	3.3685
source	.2827	.1261	2.2416	.0256	.0346	.5307
benign	-.1483	.0557	-2.6597	.0082	-.2579	-.0386
maliciou	.2873	.0583	4.9305	.0000	.1727	.4020

****

Outcome: anger

Model Summary

R	R-sq	MSE	F	df1	df2	p
.2243	.0503	1.4702	5.9688	3.0000	338.0000	.0006

Model

	coeff	se	t	p	LLCI	ULCI
constant	1.9835	.3388	5.8545	.0000	1.3171	2.6500
source	.1344	.1419	.9473	.3442	-.1447	.4135
benign	-.0276	.0627	-.4408	.6597	-.1510	.0957
maliciou	.2660	.0656	4.0563	.0001	.1370	.3950

****

Outcome: motivation

Model Summary

R	R-sq	MSE	F	df1	df2	p
.5546	.3076	1.0659	18.4934	8.0000	333.0000	.0000

Model

	coeff	se	t	p	LLCI	ULCI
constant	1.4004	.7139	1.9617	.0506	-.0039	2.8048
pride	.2834	.0754	3.7601	.0002	.1352	.4317
envy	-.0095	.0658	-.1438	.8858	-.1388	.1199
happiness	.2320	.0726	3.1964	.0015	.0892	.3747
depression	-.0710	.0797	-.8908	.3737	-.2279	.0858
anger	.0416	.0601	.6921	.4893	-.0767	.1599
source	-.1304	.1254	-1.0399	.2991	-.3770	.1163
benign	.2846	.0547	5.1990	.0000	.1769	.3923
maliciou	-.1287	.0589	-2.1848	.0296	-.2447	-.0128

******************** DIRECT AND INDIRECT EFFECTS *************************

Direct effect of X on Y

Effect	SE	t	p	LLCI	ULCI
-.1304	.1254	-1.0399	.2991	-.3770	.1163

Indirect effect of X on Y

	Effect	Boot SE	BootLLCI	BootULCI
TOTAL	-.2514	.0764	-.4189	-.1156
pride	-.1521	.0543	-.2820	-.0657
envy	-.0037	.0294	-.0654	.0539
happiness	-.0811	.0383	-.1807	-.0233
depression	-.0201	.0279	-.1022	.0149
anger	.0056	.0157	-.0106	.0642

******************** ANALYSIS NOTES AND WARNINGS *************************

Number of bootstrap samples for bias corrected bootstrap confidence intervals:

10000

Level of confidence for all confidence intervals in output:

95.00

NOTE: Some cases were deleted due to missing data. The number of such cases was:

140

The effects of achievement source on the five emotions, the effects of the emotions on moving-up motivation, the effects of trait benign and malicious envy on moving-up motivation and the direct effect of achievement source on moving-up motivation are also displayed in the Figure (coefficient and 95% CI):

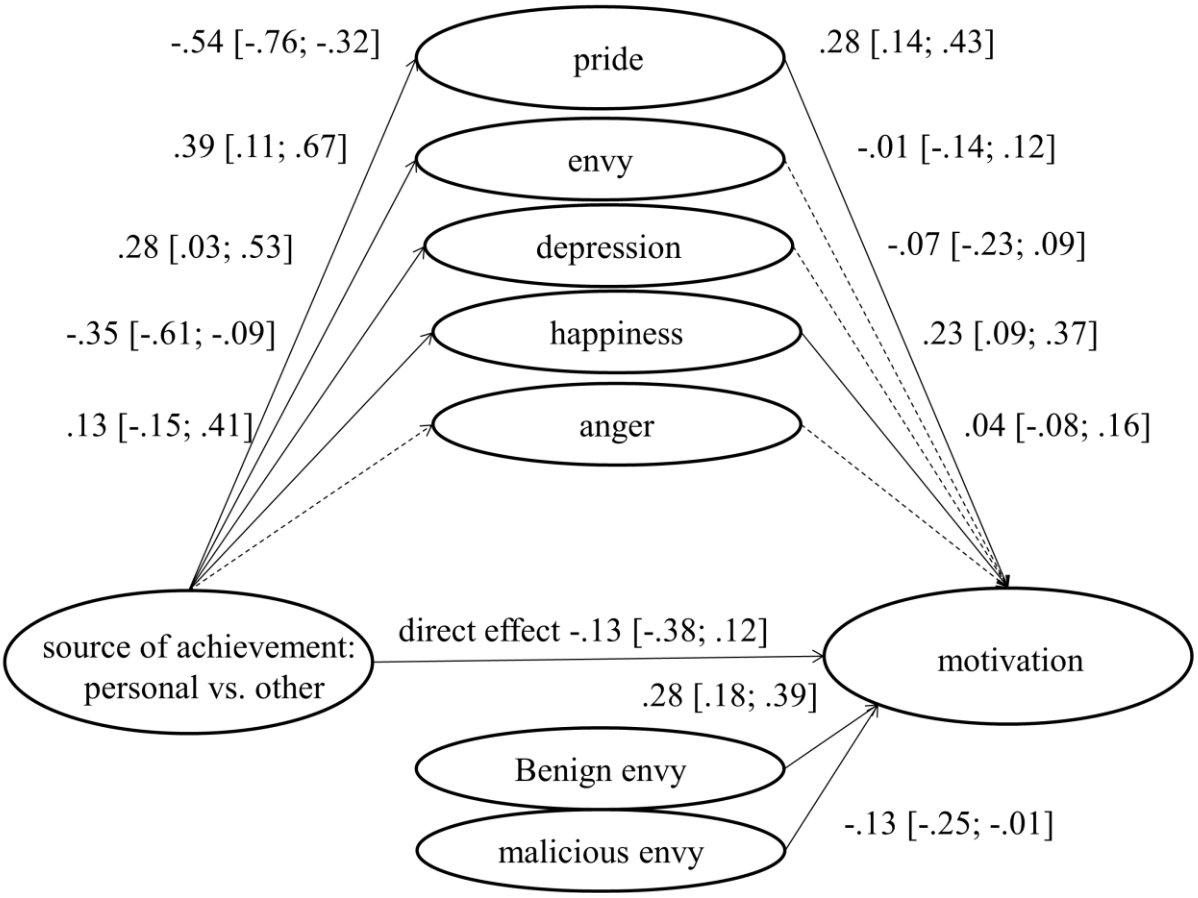

Indirect effect via pride: -.15 [-.28, -.07]

Indirect effect via happiness: -.08 [-.18, -.02]

Additionally, trait benign envy also was related to higher levels of pride and happiness and lower levels of depression (see Process output for coefficients). Malicious envy was correlated to all five emotions. The higher trait malicious envy, the more envy, depression and anger and the less pride and happiness were reported.

Data collected before the introduction of the like button

Effect on emotions. A MANOVA with achievement group (none, personal, other's) as the independent variable and pride and envy as dependent variables revealed a significant multivariate effect of achievement group, Pillai's trace = .11, $F(4,460) = 6.73$, $p < .001$, $\eta_p^2 = .09$. Respondents in the personal achievement group reported higher levels of pride ($M = 4.88$) than people in the other's achievement group ($M = 4.31$) or the control group ($M = 4.06$). The latter two groups did not significantly differ from each other (Bonferroni-protected comparisons). The univariate effect for pride was significant, $F(2,230) = 11.97$, $p < .001$, $\eta_p^2 = .03$. The univariate effect for envy was also significant, $F(2,230) = 3.47$, $p = .033$, $\eta_p^2 = .03$. Respondents in the other's achievement group reported higher levels of envy ($M = 3.31$) than people in the personal achievement group ($M = 2.82$). The no achievement group did not significantly differ ($M = 3.06$), but a Welch test revealed that envy was higher in the other's achievement group than in the other two conditions, $F(1,217.42) = 6.39$, p < .05.

Effect on motivation. A univariate analysis of variance with source of achievement as independent variable revealed a significant effect of source, $F(1, 196) = 5.15$, $p = .024$, $\eta_p^2 = .026$. Participants were more motivated when they were exposed to their personal achievement ($M = 4.38$, $SD = 1.37$) than when they were exposed to someone else's achievement ($M = 3.95$, $SD = 1.14$).

Mediation model.

We run model 4 from PROCESS (Hayes, 2013) with achievement source (personal vs. other) as independent variable, moving-up motivation as dependent variable, pride and envy as potential mediators and trait benign and malicious envy as covariates. We used 10000 bootstrapping samples.

The direct effect of achievement source (personal vs. other) on motivation was not significant, -.04, SE = .17, 95% CI [-.38; .29]. There was a significant indirect effect via pride, -.27, SE = .10, 95% CI [-.50; -.12]. The indirect effect via envy was not significant, -.05, SE = .04, 95% CI [-.17; .01]. Benign envy was positively related to motivation, .34, SE = .07, 95% CI [.19; .48]. Malicious envy in contrast was negatively related to motivation, -.20, SE = .07, 95% CI [-.35; -.06]. Trait malicious envy was negatively related to state envy, .26, SE = .09, 95% CI [.09; .43]). No other correlations with trait benign or malicious envy were significant.

Data collected after the introduction of the like button

A univariate analysis of variance with achievement group (none, personal, other's) as independent variable and moving-up motivation as dependent variable revealed a significant effect of source, $F(1, 218) = 1.78$, $p = .172$, $\eta_p^2 = .016$. Descriptively, participants were less motivated when they were exposed to someone else's achievement ($M = 4.07$, $SD = 1.21$) than when they were exposed to their personal achievement ($M = 4.41$, $SD = 1.16$) or no achievement ($M = 4.33$, $SD = 1.19$).

Exploratory analyses.

Effect of the like button. Although time of data collection had no significant effect in the analyses of variance, we nevertheless examined the use of the like button and its relationships with our variables. The like button was not used frequently; 70.6% of the respondents who saw the achievement of another person indicated that they have not liked this achievement, and 87.2% who saw their personal achievement indicated to have not received a like. Whether people have given or received a like was uncorrelated to emotions or motivation, all *r*s < .17, *ns*.
